# Dupilumab induces a rapid decrease of pruritus in adolescents: A pilot real‐life study

**DOI:** 10.1111/dth.15115

**Published:** 2021-09-02

**Authors:** Luca Mastorino, Riccardo Viola, Michele Panzone, Gianluca Avallone, Giuseppe Gallo, Michela Ortoncelli, Giovanni Cavaliere, Pietro Quaglino, Simone Ribero

**Affiliations:** ^1^ Medical Science Department, Section of Dermatology University of Turin Turin Italy

## CONFLICT OF INTEREST

The authors declare no conflict of interest.

## AUTHOR CONTRIBUTIONS

All authors contributed to the study conception and design. Material preparation, data collection and analysis were performed by Luca Mastorino. The first draft of the manuscript was written by Simone Ribero and all authors commented on previous versions of the manuscript. All authors read and approved the final manuscript.

## ETHICS STATEMENT

This study was approved by the ethic committee of the Turin University hospital (n.0070547 practice CS 2/1359). The patients signed informed consent for the publication of the images.

Dear Editor

Atopic dermatitis (AD) is a common, chronic, recurrent, pruritic skin condition affecting children, adolescents, and adults. The condition is characterized by itching, a subjective symptom that has a strong negative impact on patients' quality of life.^1^ Dupilumab is the first monoclonal antibody against moderate‐to‐severe AD in adults and adolescents (>12 years of age) approved in Europe.^1^ Dupilumab also showed safety and efficacy in the adolescent and pediatric population, with an EASI (eczema area severity index)‐75 improvement from baseline of 41.5 and 58.3%, respectively.^2^, ^3^ Dupilumab is approved for adolescents with moderate to severe AD eligible for systemic therapy since December 2020. Based on the experience acquired in adult patients and considering its proved efficacy and high safety profile, dupilumab can currently be considered for adolescents as a first‐choice systemic treatment.^4^ The treatment with dupilumab 200 or 300 mg every 2 weeks in adults and adolescents was associated with a rapid reduction in pruritus, assessed with the ppNRS (daily Peak Pruritus Numerical Rating Score) and SCORAD (SCOring Atopic Dermatitis), as reported by registration studies.^1^ Real‐life experiences on adult patients with moderate‐to‐severe AD showed a rapid pruritus control that was reached as early as 1 month of therapy with dupilumab at the standard regimen (300 mg every 2 weeks after a loading dose of 600 mg).^5^ Although data on young patients are scarce, Mareschal et al. described a case series of 4 AD adolescents treated with dupilumab and reporting a 73% reduction in SCORAD and a 45% reduction in pruritus assessed with ppNRS at week 16.^6^ Similarly, to what observed in adults, real‐life results on pruritus control in adolescents seems superior to registration studies.^5^

**FIGURE 1:**
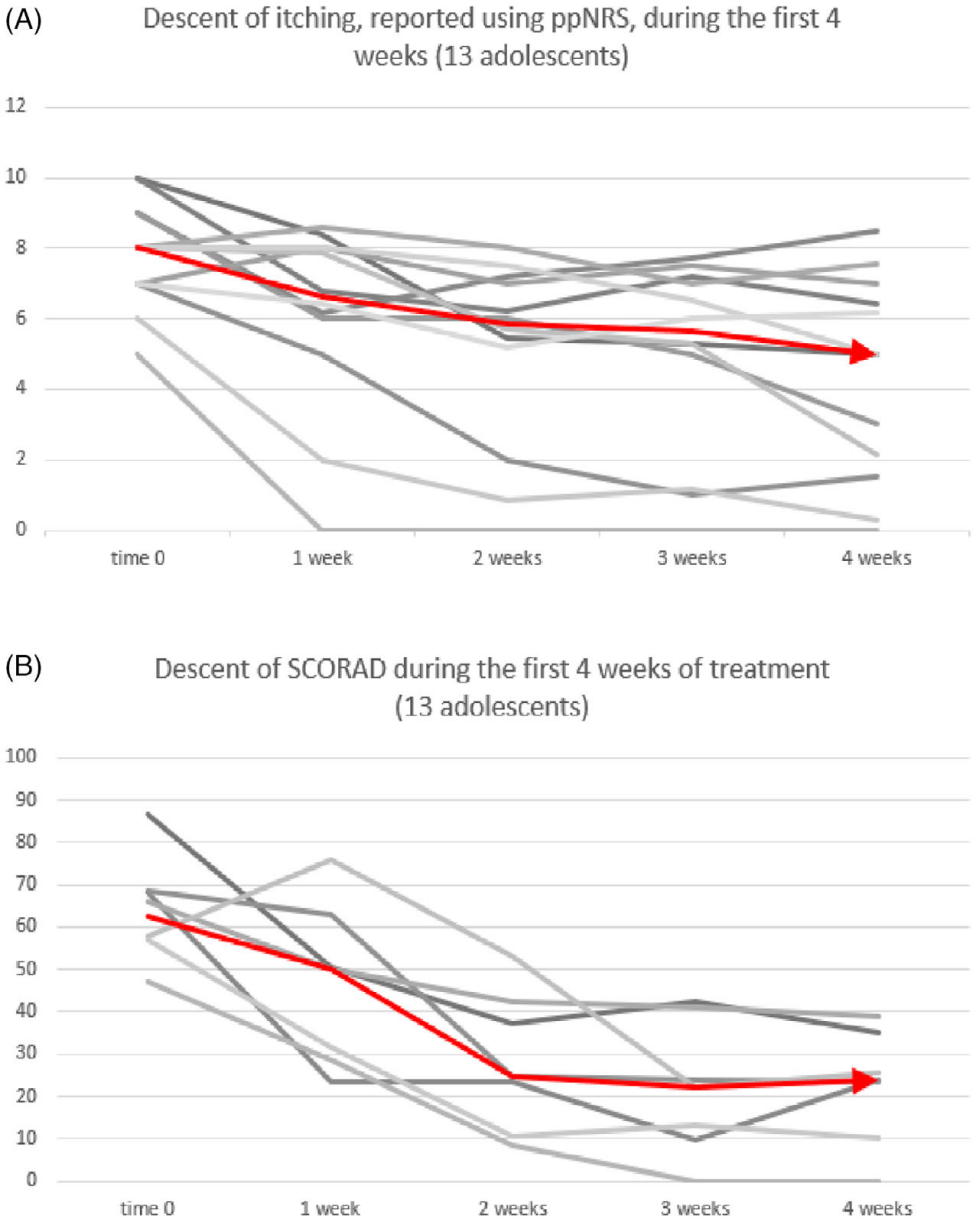
(A) The graph shows the descent curves of pruritus calculated by ppNRS score during the 4 weeks of treatment with dupilumab, the value was weekly assessed. The median descent is depicted by the red line. (B) The graph shows the descent curves of SCORAD during the first 4 weeks of treatment with dupilumab, the value was weekly assessed. The median descent is depicted by the red line

Since 2020, in our clinic we have managed 13 adolescents with moderate‐to‐severe AD using dupilumab (10 males and 3 females, mean age 15 years ranging from 13 to 17). All patients received the standard adult dose. Pruritus was the main symptom reported by our patients and it was weekly assessed by ppNRS score and SCORAD. EASI was also recorded at baseline and at week 4. The median ppNRS score of the 13 adolescents at the beginning of treatment was 8, ranging from 5 to 10. After 4 weeks of treatment, the median NRS score was 5, ranging from 8.5 to 0, with a 37.5% reduction in pruritus. No patient experienced a worsening of symptoms, while 3 patients reported a rapid reduction of pruritus already occurring at week 2. After 4 weeks, 5 of our patients reported pruritus less than 4 on ppNRS score (Figure 1A). At baseline the median SCORAD, a score that combines the size of pruritus to the severity and clinical extent of AD, was 62.7%, after 4 weeks it settled at 23.79% with a reduction of almost 40%. We also noted a rapid reduction in the median value as early as week 2 (Figure 1B).

The little difference described by SCORAD compared to the ppNRS score is probably due to the role of the progressive resolution of clinical manifestations of the disease. We observed already in the first 4 weeks an important reduction of AD manifestations in our adolescent population. The median EASI, at baseline, in our patients was 19.8, ranging from 39.9 to 10.6, after 4 weeks this value settled at 7.5, ranging from 15 to 0, with a reduction of more than 60%. 4 of 13 patients achieved EASI‐75 (30.78%; Figure S1).

Our real‐life experience confirms data reported in literature about effectiveness of dupilumab in adolescent.^7^, ^8^, ^9^ We observed a marked reduction in the median values of pruritus assessed by SCORAD and ppNRS, in association with an improvement of skin involvement. The decrease of pruritus recorded at week 4 was slightly lower than the one we had detected in adult patients (37.5 vs. 50%) in a much larger cohort.^5^ Although the sample size does not allow definitive conclusions to be drawn, dupilumab showed efficacy in the management of pruritus in our adolescent AD patients already within 1 month of treatment. Further real‐life studies with larger samples are needed to confirm these results.

## Supporting information


**FIGURE S1** The graph shows the descent curves of EASI after 4 weeks of treatment with dupilumab. The median descent is depicted by the red line.
