# Supplementary figures and images for: Dupilumab induces a rapid decrease of pruritus in adolescents: A pilot real‐life study
Source: Dermatol Ther. 2021 Sep 2;34(6):e15115. doi: 10.1111/dth.15115 (PMC9286471; doi:10.1111/dth.15115)

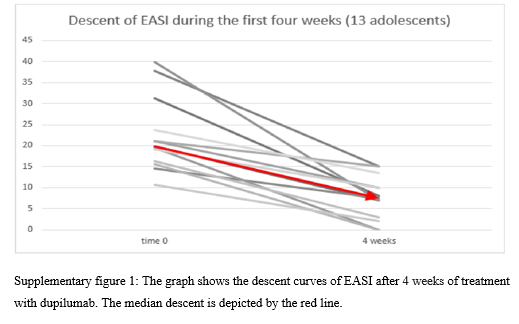

Supplement: Supplementary file 1 — FIGURE S1 The graph shows the descent curves of EASI after 4 weeks of treatment with dupilumab. The median descent is depicted by the red line. [file DTH-34-0-s001.jpeg]
